# A transcriptional biosensor to monitor single cancer cell therapeutic responses by bioluminescence microscopy

**DOI:** 10.7150/thno.63744

**Published:** 2022-01-01

**Authors:** Audrey Champagne, Pallavi Jain, Lauriane Vélot, Julie Riopel, Véronique Lefebvre, Bertrand Neveu, Frédéric Pouliot

**Affiliations:** 1 Centre de recherche du CHU de Québec-Université Laval, Québec, Canada; 2 Department of Surgery (Urology), Faculty of Medicine, Laval University, Québec, Canada; 3 Department of Laboratory Medicine (Clinical Anatomopathology), CHU de Québec-Université Laval, Québec, Canada

**Keywords:** prostate cancer, biosensor, bioluminescence microscopy, single-cell dynamic imaging, androgen receptor-axis-targeted therapy resistance.

## Abstract

When several life-prolonging drugs are indicated for cancer treatment, predictive drug-response tumor biomarkers are essential to guide management. Most conventional biomarkers are based on bulk tissue analysis, which cannot address the complexity of single-cell heterogeneity responsible for drug resistance. Therefore, there is a need to develop alternative drug response predictive biomarker approaches that could directly interrogate single-cell and whole population cancer cell drug sensitivity. In this study, we report a novel method exploiting bioluminescence microscopy to detect single prostate cancer (PCa) cell response to androgen receptor (AR)-axis-targeted therapies (ARAT) and predict cell population sensitivity.

**Methods:** We have generated a new adenovirus-delivered biosensor, *PCA3*-Cre-*PSEBC*-ITSTA, which combines an integrated two-step transcriptional amplification system (ITSTA) and the activities of the prostate cancer antigen 3 (*PCA3*) and modified prostate-specific antigen (*PSEBC*) gene promoters as a single output driving the firefly luciferase reporter gene. This system was tested on PCa cell lines and on primary PCa cells. Single cells, exposed or not to ARAT, were dynamically imaged by bioluminescence microscopy. A linear discriminant analysis (LDA)-based method was used to determine cell population sensitivities to ARAT.

**Results:** We show that the *PCA3*-Cre-*PSEBC*-ITSTA biosensor is PCa-specific and can dynamically monitor single-cell AR transcriptional activity before and after ARAT by bioluminescence microscopy. After biosensor transduction and bioluminescence microscopy single-cell luminescence dynamic quantification, LDA analysis could discriminate the cell populations overall ARAT sensitivity despite heterogeneous single-cell responses. Indeed, the biosensor could detect a significant decrease in AR activity following exposure to conventional ARAT in hormone-naive primary PCa cells, while in castration-resistant PCa patients, treatment response correlated with the observed clinical ARAT resistance.

**Conclusion:** The exploitation of bioluminescence microscopy and multi-promoter transcriptionally-regulated biosensors can aptly define the overall treatment response of patients by monitoring live single cell drug response from primary cancer tissue. This approach can be used to develop predictive biomarkers for drug response in order to help clinicians select the best drug combinations or sequences for each patient.

## Introduction

In the context of cancer, a whole range of genetic variations can take place during disease progression, such as mutation, genomic amplification, rearrangement, and alternative splicing 1-3. Tumors comprise a heterogeneous collection of cells with distinct genetic and phenotypic properties that can differentially promote progression, metastasis, and drug resistance 3-5. Moreover, during the course of disease, cancers generally become more heterogeneous with differential levels of sensitivity to treatment 3. One of the main challenges in precision medicine and predictive biomarkers' discovery is to account for this intratumoral heterogeneity 5, 6. Therefore, to optimize medical care, it is necessary to identify biomarkers that address intratumoral heterogeneity and help decide which patients to treat and which therapy is most likely to be effective.

Recent developments in single-cell sequencing technology have provided more profound insights into how therapeutic responses differ across heterogeneous genomic and transcriptomic cell states 7-9. However, static single-cell omics measurements lack the ability to decode highly dynamic cellular and molecular behaviors, like single-cell response to different stimuli 10. To better understand the therapeutic response of patient tumors, it is essential to quantitatively and dynamically measure the molecular processes that underlie cell-fate decisions in single cells 11.

A new functional single-cell assay has shown the potential of clinical samples to predict therapeutic response dynamically 12. Manalis' group defined the therapeutic susceptibility of single-cell populations from myeloma and metastatic brain cancer patient samples by measuring single-cell mass accumulation rates 13, 14. Likewise, dynamic molecular imaging of single cancer cells by bioluminescence microscopy can be used as a novel approach to image cancer cells and evaluate their response to treatment 15, 16. Indeed, the development of the recent microscope LV200 specifically designed for bioluminescence imaging with an optimized light path has dramatically increased photon detection sensitivity allowing single-cell bioluminescence activity monitoring 15-18. Moreover, bioluminescence does not require excitation from an external source, thus limiting photobleaching, background noise and auto-fluorescence, which make bioluminescent signal very sensitive and quantitative 19, 20. These single cell analysis approaches have the potential to provide a more comprehensive picture of the heterogeneous dynamics in therapeutic response and the emergence of resistance. This is especially the case in the context of prostate cancer (PCa), where 60% of metastatic castration-resistant PCa (mCRPC) patients harbored more than one gene alteration associated with resistance, expressed in different single cell, like mutations of the androgen receptor (AR) locus 21. We have shown that bioluminescence microscopy, in combination with the adenoviral delivery of a two-step transcriptional amplification system (TSTA) driven by a modified prostate-specific antigen (PSA)-promoter (*PSEBC*), named *PSEBC*-TSTA, can monitor single-cell response to androgen receptor-axis-targeted therapies (ARAT); the main therapeutic target in PCa 15. These new single-cell technologies enable the characterization of the cellular phenotype resulting from multiple factors influencing cellular response.

*PCA3* long non-coding RNA is a unique PCa oncogene and biomarker that is amplified 60-fold in PCa when compared to non-PCa epithelial cells 22. We have previously exploited the PCa specificity of the *PCA3* promoter to drive a new amplification system, the three-step transcriptional amplification system (3STA), to image primary PCa cells by bioluminescence. We have shown that the *PCA3* promoter was overactive in primary PCa biopsies when compared to benign prostate tissue 23. Interestingly, the development of a urine PCa screening test based on PCA3 long non-coding RNA expression levels and recent studies have demonstrated that PCa cells are found in the urine of patients after prostatic massage 22, 24, 25. Apart from *PCA3* promoter, the *PSA* promoter is also active in these cells. However, the *PSA* promoter alone cannot be used as a dynamic biomarker of response to ARAT since benign prostate cells can be found in biopsies or in the urine.

In this study, we describe a novel biosensor that is based on a transcriptional imaging method, which combines the specificities of *PSEBC* (androgen-sensitive) and *PCA3* (PCa-specific) promoters and dynamic imaging capabilities of bioluminescence microscopy. We show that this method can monitor single-cell response to ARAT and predict cell population drug sensitivity.

## Materials and methods

### Plasmid and adenoviral constructions

#### Generation of stop cassette plasmids

Bovine growth hormone polyA sequence 26 along with the LoxP on its flanking ends was synthesized from GenScript (Piscataway, NJ, USA) and the SV40 stop cassette was obtained from plasmid pBS302 (Addgene, Watertown, MA, USA) 27. Stop cassette along with the LoxP sites at its ends were inserted into pGL3-promoter vector (Promega, Madison, WI, USA).

#### Generation of modified Cre recombinase cDNAs

Cre recombinase gene was amplified from plasmid pMC-CreN 28 (kindly provided by Dr Jean Charron, Laval University, Canada) in two fragments to insert the intron. The intron *BGH-Ig* was amplified from plasmid pIC 27), and the intron *Prm2* and intron *Prm2*-AG were amplified from genomic DNA of mice. Primers used for amplification are described in Table S1. CMV and PCA3 promoters were amplified by PCR as previously described 23. Four-fragment ligation (promoter, Cre first fragment, intron and Cre second fragment) into the plasmid pENTR-L5R2 backbone was done using Gibson Assembly® cloning kit (New England Biolabs, Ipswich, MA, USA) to obtain pENTR-CMV-Cre and pENTR-PCA3-Cre.

#### Construction of plasmids with LoxP sites

*SV40* promoter sequence, LoxP sites flanking the stop cassette and GAL4VP16 sequence were synthesized (GenScript). Plasmid pENTR-L1R5-GAL4RE-Luc 23 was digested using SalI restriction site and the synthesized fragment was inserted into the plasmid using In-Fusion HD cloning kit (Takara Bio USA, Mountain View, CA, USA) into different orientations. The prostate-specific* PSEBC* promoter was amplified from pENTR-*PSEBC*-GAL4VP16 15 using primers described in Table S1. *SV40* promoter was digested out with SalI and BsiWI restriction sites and replaced by* PSEBC* using In-Fusion HD cloning kit (Takara).

#### Adenoviral constructions

Adenovirus expressing *PCA3*-3STA, *PSEBC*-TSTA and *SV40*-Luc were generated as previously described 23. To obtain the multi-promoter integrated two-step transcriptional amplification system, *PCA3*-Cre-*PSEBC*-ITSTA, in a single backbone containing the Cre recombinase and the TSTA system with the stop cassette, the above constructed pENTR-L1R5 and pENTR-L5R2 backbone plasmids were subcloned into pAd-pL-DEST by LR cloning with LR clonase II Plus enzyme (Invitrogen, Carlsbad, CA, USA). Adenoviral backbones containing plasmids were transfected into 293A cells for the viral production. Amplified virus particles were column purified using Adeno-X™ Maxi purification kit (Takara) and stored in buffer A195 after buffer exchange 29. Titration for each of the viruses was done using Adeno-X™ Rapid Titer Kit (Takara).

### Cell line culture

22Rv1, LNCaP, LNCaP-LN3, LNCaP-PRO5, V16D, MR42D, MR49F, CAMA-1 and ZR-75-1 were cultured in RPMI-1640 media containing 10% fetal bovine serum (FBS) (VWR, Radnor, PA, USA). LAPC4 was cultured in DMEM media (Wisent Bio Products, ST-BRUNO, QC, Canada) containing 10% FBS. PC-3, DU145, SW780, SW1710, RT4, MD-MB-231 and MCF-7 were cultured in eMEM (Wisent Bio Products) media containing 10% FBS. All the cells were incubated in a 37 °C incubator that provided 5% CO_2_. Cells were passaged after the confluence reached 80-90%.

### Adenoviral infection and treatment experiments for luciferase assays

Cancer cell lines were seeded in 24-well plates as follow: 22Rv1, LNCaP, LNCaP-LN3, LNCaP-PRO5 at 1 x 10^5^ cells/well; V16D, MR42D, MR49F, CAMA-1, ZR-75-1, MD-MB-231 and MCF-7 at 5 x 10^4^ cells/well; PC-3, DU-145, SW780, SW1710 and RT4 at 8 x 10^4^ cells/well. Twenty-four hours after seeding, adenoviruses were transduced at a multiplicity of infection (MOI) of 2. Seventy-two hours after infection, cells were lysed, and a luciferase assay was performed as described (Promega, Madison, WI, USA). Protein content was estimated by adding 250 μl of Bradford reagent (ThermoFisher Scientific, Waltham, ON, Canada) to 3 μl of total lysate. Absorbance was then read using an Infinite F50 absorbance microplate reader (TECAN, Mannedorf, Switzerland) at 595 nm. *SV40*-Luc virus was infected in parallel to normalize for infection efficiency between different cell lines. For androgen sensitivity assessment, cells were treated with 10 nM dihydrotestosterone (DHT) (Toronto Research Chemicals, Toronto, ON, CA) and 10 μM Bica (Sigma-Aldrich, St.Louis, MO, USA) in 10% charcoal-treated FBS (FBS-CT) (Wisent Bio Products), 24 h post-infection. Luciferase assays were performed after 48 h of treatment.

### Transfection experiments for luciferase assays

LAPC4 (1 x 10^5^ cells/well) were seeded in 24-well plates. The following day, 100 ng of each plasmid was transfected into the cells along with 60 ng of pRL-null (Promega) using lipofectamine 2000 (Invitrogen). Seventy-two hours after transfection, the cells were lysed using passive lysis buffer and a luciferase assay was performed as described (Promega).

### RT-qPCR technique

The 22Rv1 cells were infected with adenovirus expressing or not Cre recombinase. Cells were washed with PBS and trypsinized at each of the time points (6, 24, 48, 72, 96 h). Viral DNA was isolated from the infected cells using QIAmp® viral DNA isolation kit (Qiagen, Hilden, Germany). RT-qPCR reaction was performed with TaqMan probes (Applied Biosystems, Waltham, MA, USA) using two primer sets, one within the firefly luciferase (Luc) gene used as internal control (primer set 1) and one within the stop cassette to determine the cleavage (primer set 2) (Table S1). Standard curves for both the primer sets were determined using original plasmids as the template. Isolated viral DNA copy number at each time point was extrapolated on the standard curves.

### Cell lines adenoviral infection and treatment for dynamic bioluminescence assays and imaging

LNCaP, LAPC4 and 22Rv1 cells (2,000 cells/well) were seeded in a 384-well black plate (Greiner Bio-One North America Inc., Monroe, NC, USA) in RPMI-1640 with 10% heat-inactivated FBS-CT and 1 nM DHT. The cells were then transduced with 1 x 10^5^ infectious viral particles (ivp) of *PCA3*-Cre-*PSEBC*-ITSTA adenovirus per well. The plate was kept on a shaker overnight and incubated at 37 °C. Seventy-two hours after infection, media was removed to leave 10 μl at the bottom of the wells. Ten microliters of Matrigel^TM^ Matrix High Concentration (Corning, Corning, NY, USA) diluted at 40% in cold RPMI-1640 (with 10% heat-inactivated FBS-CT and 1 nM DHT) was added in each well. The plate was rapidly centrifuged at 225 g for 3 min and incubated for 30 min at 37 °C in 5% CO_2_. Following incubation, 30 μl of RPMI-1640 with 10% heat-inactivated FBS-CT and 1 nM DHT was added to each well and, in the presence of 3.5 mM of D-luciferin (Caliper Lifesciences, Hopkinton, MA, USA), wells were then read with a TriStar LB 941 (20 seconds exposure; Berthold, Bad Wildbad, Germany) or imaged. To further determine response to treatment for isolated cells, media over the Matrigel^TM^ layer was replaced with media containing 1 nM DHT, 1 nM DHT + 10 μM Bica or 1 nM DHT + 10 μM Enza (MedChem Express, NJ, USA) and reading or imaging was done 48 h later. D-luciferin (3.5 mM) was added 20 min before each imaging timepoint.

### Patient samples

Samples were collected with ethical permission from the Institutional Review Board of the CHU de Quebec Hospital, Quebec, QC, Canada (A14-06-1976 and A12-12-1076). All patients gave written consent for their tissue to be used for research. Primary prostate samples were obtained from needle biopsy cores (18 G, 17 mm) of radical prostatectomy of naive patients. Sextants harboring adenocarcinoma on a previous transrectal biopsy were targeted. Urine samples were collected from mCRPC patients with primary tumor and established clinical status.

### Isolation, infection and treatment of PCa cells isolated from urine samples

Ten milliliters of urine were collected post-digital rectal examination. Sampling was done twice for patient 8, the first and second sampling were done when the patient was on enzalutamide and bicalutamide, respectively. Urine samples were centrifuged at 400 *g* for 10 min at 4 °C. Then supernatant was carefully removed, leaving 2 ml of the sample. The pellet was re-suspended gently in 30 ml of washing buffer (1X PBS + 2% FBS). The solution was centrifuged again at 200 *g* for 10 min at room temperature (RT). Supernatant was carefully discarded and gently resuspended in 30 ml of media (RPMI-1640 with 10% heat-inactivated FBS-CT with 1 nM DHT, penicillin and streptomycin). Centrifugation was repeated at 200 *g* for 10 min at RT. Supernatant was carefully removed leaving behind 400 µl of media containing the isolated cells and 50 μl was seeded in each well of a 384-well plate. The samples were then infected with 5×10^5^ ivp of* PCA3*-Cre-*PSEBC-*ITSTA adenovirus per well. The plate was kept on a shaker overnight and incubated at 37 °C. Seventy-two hours after infection, Matrigel^TM^ layer and media were added to wells as described above and the wells were imaged in the presence of D-luciferin. Next, media over the Matrigel^TM^ layer was replaced with the appropriate treatment media containing 1 nM DHT, 1 nM DHT + 10 μM Bica or 1 nM DHT + 10 μM Enza. Imaging was repeated 48 h post-treatment. Bioluminescence imaging was done 20 min after adding 3.5 mM of D-luciferin.

### Dissociation, infection and treatment of primary PCa samples

The biopsy samples were washed three times with HBSS-Ca^2+^-Mg^2+^ (Wisent Bio Products), cut into 1 mm^2^ fragments and incubated overnight at 37 °C (5% CO_2_) with shaking in Advanced DMEM/F12 media (ThermoFisher Scientific) complemented with 1X GlutaMAX^TM^ (ThermoFisher Scientific), 1X Hepes (Sigma-Aldrich) and 1X Primocin (ThermoFisher Scientific) with 100 U/ml of type II Collagenase (ThermoFisher Scientific) and 0.005 MU/ml of DNAse (Milipore, Burlington, MA, USA). The next day, samples were dissociated by vigorous pipetting and incubated at 37 °C (5% CO_2_) with shaking for another 2 h. Cell suspensions were washed in a 15 ml tube with 10 ml of 1X PBS and centrifuged at 500 *g* for 10 min. Supernatants were gently removed, and the cells were resuspended in Accumax^TM^ solution (Sigma-Aldrich) and incubated for 20 min at 37 °C with frequent agitation. The cells were washed with 10 ml of 1X PBS and resuspended in RPMI-1640 media containing 10% heat-inactivated FBS-CT and 1 nM DHT before seeding in a 384-well plate at a concentration of 2,000 viable cells/well. The cells were then infected with 10^6^ ivp of* PCA3*-Cre-*PSEBC-*ITSTA adenovirus per well. The plate was incubated at 37 °C (5% CO_2_) for 72 h including rotative agitation for the first 16 h. The Matrigel^TM^ layer and media were then added to the wells as describe above and the wells were imaged in the presence of D-luciferin. Next, media over the Matrigel^TM^ layer was replaced with the appropriate treatment media containing 1 nM DHT, 1 nM DHT + 25 μM Bica or 1 nM DHT + 35 μM Enza. Imaging was repeated 72 h post-treatment.

### Bioluminescence microscopy imaging

Dynamic bioluminescence microscopy was performed using an LV200 microscope (Olympus, Tokyo, Japan) as previously described 15. The bioluminescent LV200 microscope is equipped with an EM CCD camera (Andor Ixon 897) and an incubation chamber with temperature control, humidity and gas flow to keep the prostate cells at 37 °C under 5% CO_2_. Briefly, each bioluminescence imaging was performed 20 min after adding D-luciferin at 3.5 mM using Olympus UPLSAPO 40X objective (a non-immersive lens with a numerical aperture of 0.95, a working distance of 0.18 mm and a correction collar from 0.11 to 0.23 mm) with exposure times of 20 sec per field of view (FOV) as previously described 15. The threshold for AR active cells was defined as luminescent signals over background. Because the luminescent signal is the result of an enzymatic reaction that requires ATP for conversion of D-luciferin substrate into oxyluciferin, only live cells expressing the reporter gene would produce light 30. Data analysis and process design for automated image capture were achieved using the CellSens software (Olympus).

### Immunofluorescence

Patient cell samples were fixed after the second bioluminescence imaging, while cell lines were fixed 24 h after seeding. Cells were fixed using 4% paraformaldehyde, then permeabilized with 0.5% triton-X 100 in PBS for 10 min. The cells were washed in PBS, blocked with 5% bovine serum albumin (BSA), and then incubated for 1 h at RT with primary antibodies against: alpha-Methylacyl-CoA racemase (AMACR, 1:50) (ab93045, Abcam, Toronto, ON, Canada), Nucleolin (1:200) (ab136649, Abcam,) or NKX3.1 (1:100) (AES0314, ^MJS^BioLynx Inc., Brockville, ON, Canada). Primary antibodies were diluted in PBS-2% BSA. After washing cells three times with PBS containing 0.025% Tween 20, cells were subsequently treated with secondary Anti-mouse IgG Alexa Fluor 488, 1:500 (4408S, New England Biolabs) and Anti-Rabbit IgG Alexa Fluor 594, 1:200 (A11012, Thermofisher Scientific) diluted in PBS-2% BSA for 1 h at RT. After washing cells three times with PBS containing 0.025% Tween 20, nuclei were co-stained for 5 min with DAPI, washed again, and cells were retained for fluorescent imaging.

### Statistical analysis

Linear discriminate analyses (LDA) were performed using Microsoft Excel 16.0 and XLSTAT Addinsoft version 2020.1.3.65325 (Addinsoft Inc., New York, U.S.). LDA factor scores and ROC curves were obtained from these analyses. Comparison of areas under the ROC curve (AUC) was done with the DeLong method 31. Statistical analyses were performed using GraphPad Prism (La Jolla, CA, USA). Bar graphs were expressed as mean ± standard deviation (SD). Data were compared by one sample t-test, paired and unpaired Student's t-test, one-way or two-way analysis of variance (ANOVA). Post hoc Bonferroni, Tukey or Dunnett tests were performed where significant interactions were observed in ANOVAs. Significance was established as p ≤ 0.05 (*), 0.01 (**), 0.001 (***), and 0.0001 (****).

## Results

### Integration of PCA3 and PSEBC promoter specificities as a single output to image PCa cell antiandrogen response

**Figure 1:**
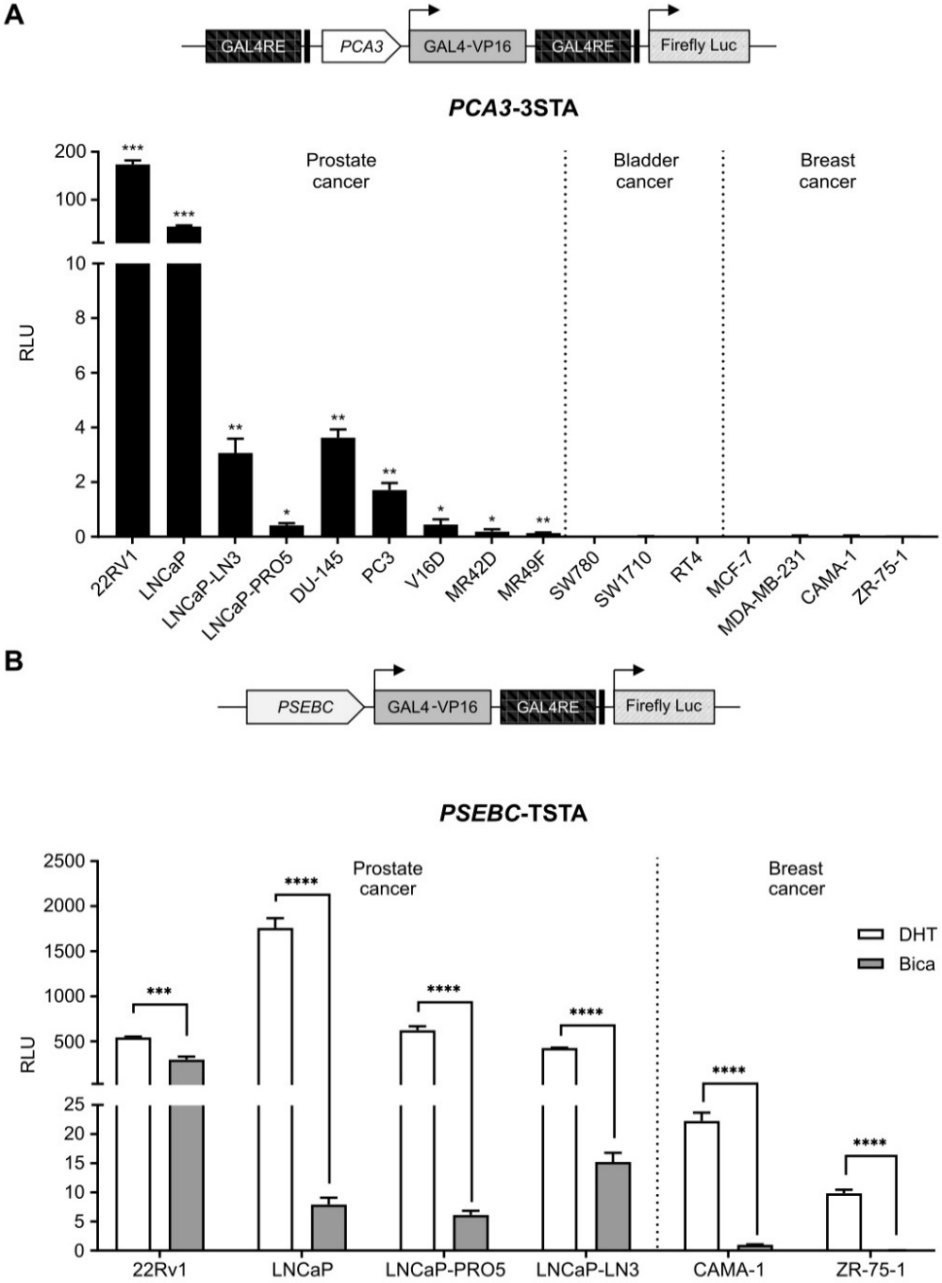
** The *PCA3* promoter is prostate cancer-specific while *PSEBC* promoter is androgen responsive. (A)** The *PCA3* promoter is highly prostate cancer (PCa)-specific. **(B)** The *PSEBC* promoter is active in androgen responsive cell lines. Luciferase assay of prostate cancer cells, bladder cancer cells and breast cancer cells infected with *PCA3*-3STA or *PSEBC*-TSTA for 72 h. In case of *PSEBC*-TSTA, 24 h post-infection, media was replaced with media containing DHT or Bica. The luciferase activity was first normalized by protein content in each well and then normalized according to the average of luciferase activity driven by *SV40* promoter (*SV40*-Luc) in each cell line (RLU = (luciferase activity/μg protein) ÷ (SV40 luciferase activity/μg protein)). Data represents mean of triplicates ± standard deviation (SD). Data were compared by one sample t-test (A) and unpaired Student's t-test (B). AR: androgen receptor; Bica: bicalutamide; DHT: dihydrotestosterone; RLU: relative light unit.

To allow identification of PCa cells sensitive to ARAT, we needed to design a system that was both PCa-specific and that enabled imaging of AR transcriptional activity. When the *PCA3* promoter driving a strong three-step amplification system (3STA) was tested in PCa and non-PCa cell lines, it was highly active in PCa cells but not in breast or bladder cancer cell lines (Figure 1A); it was also not regulated by androgens 23. Contrary to the *PCA3* promoter, the *PSA* chimeric promoter, *PSEBC,* was highly androgen responsive in AR-expressing PCa cell lines and it was also active in AR-expressing breast cancer cells such as CAMA-1 and ZR-75-1 (Figure 1B).

**Figure 2:**
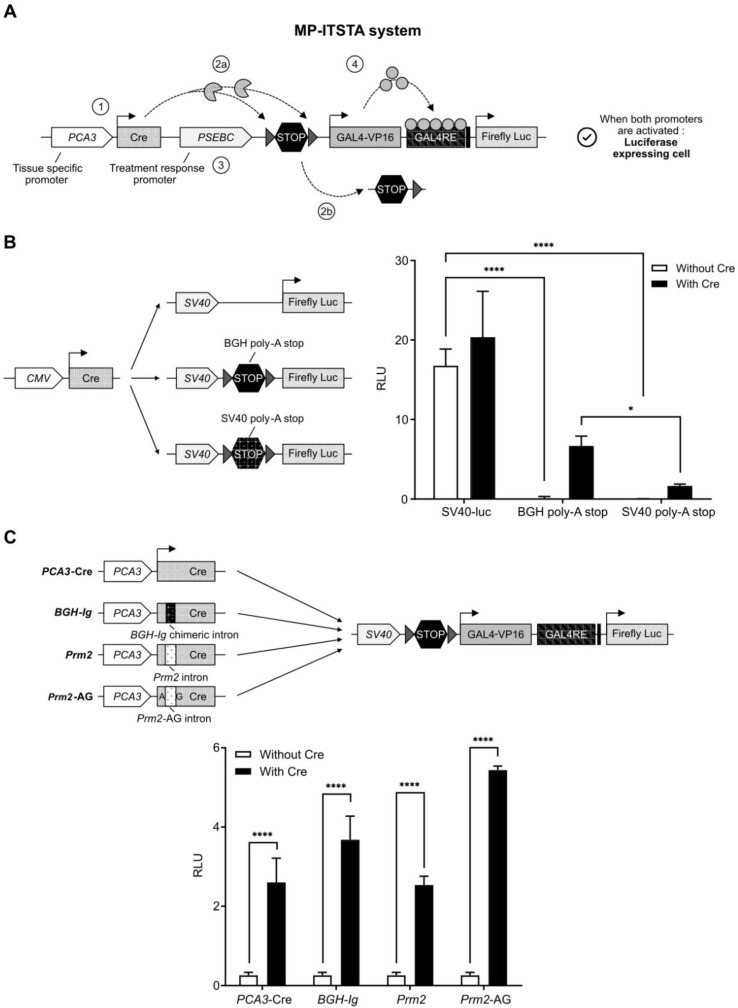
** A new biosensor system designed to combine the specificity of two promoters for driving the expression of a single reporter gene after TSTA transcriptional amplification. (A)** Activation scheme for multi-promoter integrated TSTA (MP-ITSTA) system driven by the *PCA3* and *PSEBC* promoters (*PCA3*-Cre-*PSEBC*-ITSTA). **(B)** BGH poly-A stop cassette efficiently inhibited the expression of luciferase and gave better reactivation in the presence of Cre compared to SV40 poly-A stop. **(C)** Insertion of the chimeric human intron within Cre recombinase without affecting the expression of firefly luciferase. Luciferase assay of LAPC4 cells co-transfected with plasmids as described above along with pGL3-renilla-null for 72 h. Firefly luciferase activity was normalized over renilla activity (RLU = firefly luciferase activity/renilla luciferase activity). Data represents mean of triplicates ± standard deviation (SD). Data were compared by two-way analysis of variance (ANOVA) followed by Bonferroni's multiple comparisons tests (B) and unpaired Student's t-test (C). *BGH-Ig:* human β-globin and immunoglobulin*;* BGH poly-A: Bovine growth hormone poly-A;* Prm2:* mice protamine; *Prm2*-AG: Mice protamine with AG splice site; RLU: relative light unit.

To exploit the combined potential of* PCA3* and* PSEBC* promoters as a single output (PCa specificity and AR activity monitoring), we developed the multi-promoter integrated two-step transcriptional amplification system (MP-ITSTA) (Figure 2A). MP-ITSTA utilizes the site-specific recombination ability of Cre recombinase to specifically remove the DNA fragment between two LoxP sites. The system consists of 4 major steps: 1) the activation of the first promoter leads to the production of Cre recombinase; 2) the Cre recombinase then identifies the LoxP sites and cleaves the DNA fragment between them (which contains a stop cassette); 3) a second promoter is activated and the GAL4VP16 protein is produced; 4) the GAL4VP16 fusion protein binds to GAL4RE upstream of the reporter gene and amplifies promoter-driven expression. Therefore, this system is designed to combine the specificity of two promoters in order to drive the expression of a single reporter gene after TSTA transcriptional amplification (Figure 2A).

As a first step for the development of the MP-ITSTA system, we inserted a sequence that would completely inhibit the Luc expression in the absence of Cre recombinase between the LoxP sites, namely the stop cassette. It has been shown that recombination efficiency between two LoxP sites on the same DNA molecule is dependent on the distance between them. The minimum distance required between two sites to allow recombination is 82 base pairs long 32. Therefore, we tested the ability of the bovine growth hormone (BGH) or Simian Virus 40 (SV40) polyadenylation (poly-A) sequences inserted between the LoxP sites (as a stop cassette) to inhibit the ubiquitous *SV40* promoter (Figure 2B). As shown, both sequences could block the *SV40* promoter-driven firefly luciferase gene in the absence of Cre. Upon co-transfection with the plasmid expressing Cre recombinase under ubiquitous* CMV* promoters, the BGH poly-A stop led to a better reactivation of the system giving 4.04-times higher Luc signal compared to the SV40 poly-A stop (Figure 2B). Following this result, we used the BGH poly-A stop sequence for our next experiments.

Adenovirus containing both the Cre recombinase and the LoxP in a single backbone were generated. In concordance with earlier studies 27, it was not possible to amplify adenoviral backbone plasmids containing both the wild-type Cre recombinase and the LoxP sites. This was secondary to the leaky expression of wild-type Cre recombinase causing non-specific cleavage of LoxP sites in prokaryotic bacterial systems. To inhibit bacterial expression of Cre, an intron was inserted 465 nucleotides downstream of the Cre recombinase start site; the absence of post-transcriptional splicing machinery in *E. coli* would prevent the expression of functional Cre and allow adenoviral DNA amplification. We tested three intron sequences inserted in the Cre cDNA: 1) a human *BGH-Ig* chimeric intron (5′-donor site from the first intron of the human β-globin and the branch and 3′-acceptor site from the intron located between the leader and body of an immunoglobulin gene heavy chain variable region 27), 2) the mice protamine 2 gene (*Prm2*) intron 33 and 3) the modified *Prm2* containing eukaryotic splice site (AG). As shown in Figure 2C, all three intron sequences, when inserted in the Cre cDNA, did not affect the expression of Luc when compared to wild-type Cre after plasmid transfection. However, only the Cre recombinase containing chimeric human intron allowed amplification of adenoviral backbones in bacteria. Therefore, the *BGH-Ig* chimeric human intron was used in all our MP-ITSTA constructs.

**Figure 3:**
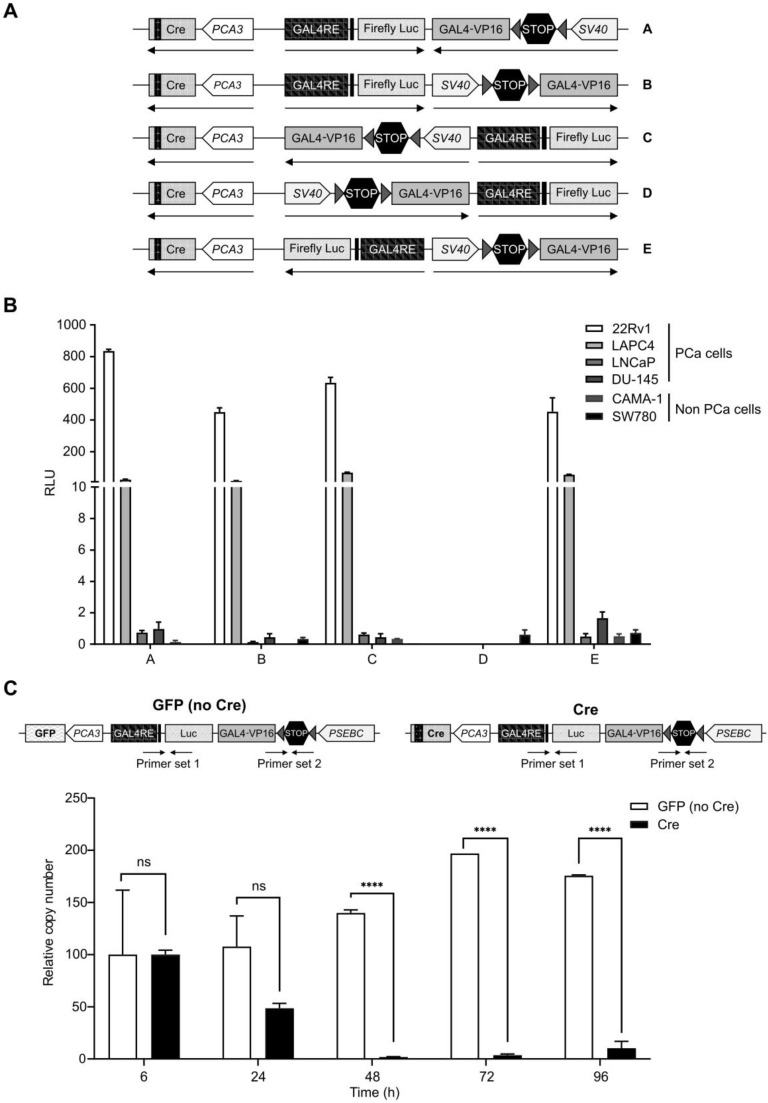
** Characterization of the best MP-ITSTA conformations for prostate cancer-specific expression and validation of the loxP site excision by Cre. (A)** Scheme of non-replicative reporter adenoviruses. **(B)** Amplification provided by orientation A was highest and had the least leaky expression in non-PCa cells. Androgen-sensitive prostate cancer cells (22Rv1, LAPC4), androgen receptor-deficient prostate cancer cells (DU-145), breast cancer cells (CAMA-1) and bladder cancer cells (SW780) were infected with non-replicative adenovirus with the above-mentioned orientations at 2 MOI. Seventy-two hours after infection, the cells were lysed, and luciferase assay was performed. Relative luciferase activity was normalized over total protein and then normalized over luciferase activity driven by SV40 promoter (*SV40*-Luc) in each cell line (RLU = (luciferase activity/μg protein) ÷ (SV40 luciferase activity/μg protein)). **(C)** *PCA3*-dependent Cre expression led to efficient deletion of DNA between the loxP sites. The 22Rv1 cells were infected with replication-deficient adenovirus expressing the firefly luciferase gene under control of *PCA3*-Cre-*PSEBC*-ITSTA (with Cre) or *PCA3*-GFP-*PSEBC*-ITSTA (no Cre) at 2 MOI. Cells were harvested at the indicated time points and viral DNA was isolated. Quantitative real-time PCR was done for the isolated DNA using two sets of primers: Primer set 1 amplifying luciferase as internal control and Primer set 2 amplifying a region within the stop cassette. Relative copy number (RCN) = ((copy number of stop cassette/copy number of luciferase)/RCN at 6 h × 100). Each data represents mean of triplicates ± standard deviation (SD) of a representative experiment. Data were compared by unpaired Student's t-test. DHT: dihydrotestosterone; PCa: prostate cancer; RCN: Relative copy number; RLU: Relative light unit.

Additionally, the biosensor that we describe in this manuscript consists of three promoters: *PCA3*, *PSEBC* and GAL4RE minimal-TK promoter, expressing different genes. It is known that having two promoters very close to one another causes steric hindrance and also competition in binding of transcription factors, thereby reducing gene output 34-36. To account for these factors, we had to find the optimal relative orientation of each component of the MP-ITSTA (promoters, amplifier, reporter). We compared Luc activities when the activator and amplifier cassette were in several orientations. For these experiments, the *SV40* promoter was driving TSTA and the *PCA3* promoter was driving Cre expression (Figure 3A). After testing the system in 22Rv1, LAPC4, DU-145 (PCa), CAMA-1 (breast cancer) and SW780 (bladder cancer) cell lines, we observed that orientation A provided the highest reporter gene signal while also being specific to PCa cells due to *PCA3*-driven Cre expression (Figure 3B).

As a final step, we determined if the Cre recombinase levels produced by *PCA3* weak promoter was sufficient to recombine all the LoxP sites. Quantitative PCR using primer sets within the stop cassette or firefly luciferase gene as an internal control showed that* PCA3*-driven Cre recombinase could remove more than 98% of the stop cassette after only 48 h. As a control, a TSTA virus expressing green fluorescent protein (GFP) instead of Cre recombinase did not show any cleavage of the stop cassette and DNA copy number of viruses remained stable over time (Figure 3C).

### PCA3-Cre-PSEBC-ITSTA system is specific to PCa cells and can measure the transcriptional activity of the androgen receptor

**Figure 4:**
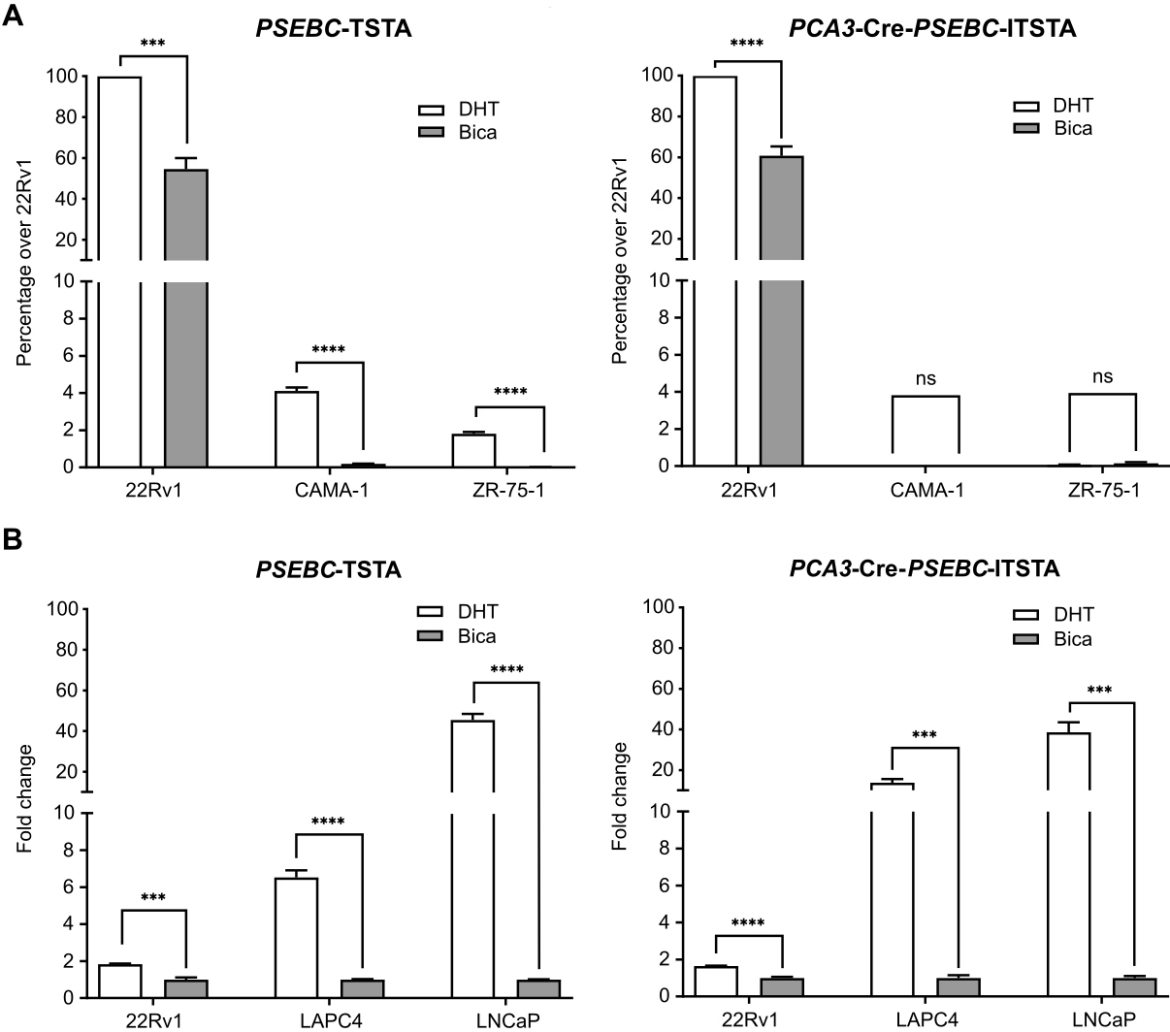
***PCA3*-Cre-*PSEBC*-ITSTA shows activity specifically in androgen receptor responsive prostate cancer cells giving an induction comparable to *PSEBC*-TSTA. (A)** *PCA3*-Cre-*PSEBC*-ITSTA is active only in AR sensitive prostate cancer cells. **(B)** Levels of induction seen with AR agonist DHT with *PCA3*-Cre-*PSEBC*-ITSTA is similar to *PSEBC*-TSTA. Luciferase assay of AR responsive prostate cancer cells (22Rv1, LAPC4, LNCaP) and AR responsive breast cancer (ZR-75-1 and CAMA-1) cells infected with *PSEBC*-TSTA or *PCA3*-Cre-*PSEBC*-lTSTA and treated with DHT or Bica for 48 h. The luciferase activity was first normalized by protein content in each well and then normalized according to the average of luciferase activity driven by *SV40* promoter (*SV40*-Luc) in each cell line (RLU = (luciferase activity/μg protein) ÷ (SV40 luciferase activity/μg protein)) and represented as relative activity over 22Rv1 in the case of **(A)** or relative activity over bicalutamide in the case of **(B)**. The data represents mean of triplicates ± S.D. Data were compared by unpaired Student's t-test. AR: androgen receptor; Bica: bicalutamide; DHT: dihydrotestosterone; RLU: relative light unit.

The *PCA3* promoter was shown to be PCa-specific, while the *PSEBC* promoter was not, but it can monitor response to androgen deprivation therapy (Figure 1). Thus, incorporation of these two promoters together in the multi-promoter integrated system could theoretically monitor androgen deprivation therapy response in PCa cells harvested from patients. We first tested the ability of* PCA3*-Cre-*PSEBC*-ITSTA to signal specifically in PCa cells.* PCA3*-Cre-*PSEBC*-ITSTA generated Luc activity was more than 1500-times higher in 22Rv1 PCa cells when compared to non-prostatic CAMA-1 or ZR-75-1 cells. By contrast, this ratio was only 24-times higher when the* PSEBC*-TSTA system was used (Figure 4A). In fact, *PCA3*-Cre-*PSEBC*-ITSTA not only restricted the expression of Luc to PCa cells but it also kept the sensitivity of the system to the AR agonist (DHT) to levels comparable to that obtained when using the *PSEBC* promoter alone (Figure 4B).

### Dynamic bioluminescence imaging of single cells allows characterization of heterogeneous androgenic response in AR-active PCa cell lines

Before undertaking bioluminescence microscopy studies, we have tried to dynamically monitor our biosensor luminescence signal variation after ARAT treatment using a standard bioluminescent plate reader. Unfortunately, this technique could not detect a significant signal variation after ARAT treatment nor different response patterns between ARAT sensitive and insensitive cell lines (Figure S1). Consequently, we have tested bioluminescence microscopy technology to monitor single-cell response to ARAT therapy 15. Bioluminescence microscopy allows same-cell reporter activity quantification and tracking before and after treatment. This same-cell normalization is needed to compensate for cell-to-cell differences in viral transduction, viability in culture or transcriptional activity.

**Figure 5:**
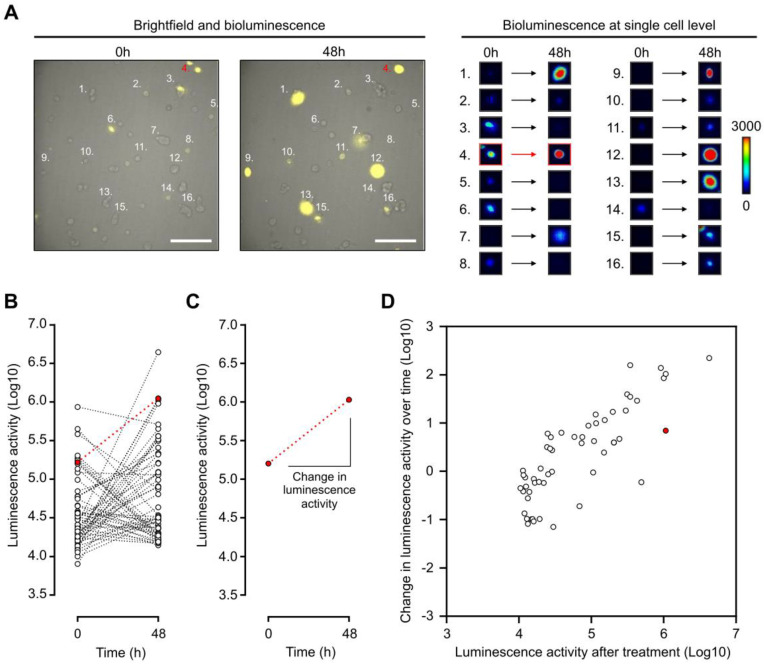
** Dynamic bioluminescence imaging of single cells expressing *PCA3*-Cre-*PSEBC*-ITSTA allows characterization of heterogeneous androgenic response in single prostate cancer cell line population. (A)** Bioluminescence imaging of LAPC4 cells expressing *PCA3*-Cre-*PSEBC*-ITSTA treated with DHT for 48 h. Right panel shows examples of bioluminescence signal intensity of 16 single cells over time. Scale bar represents 200 μm. **(B)** Plot of dynamic monitoring of luminescence activity for LAPC4 cells expressing *PCA3*-Cre-*PSEBC*-ITSTA treated with DHT for 48 h. **(C)** Change in the luminescence activity of the cell highlighted in red. Change in luminescence activity is calculated from the slope between the two-time data. **(D)** Change in luminescence activity over time calculated from each individual cell is plotted with respect to luminescence activity after treatment of each cell. Cell number 4 highlighted in **(A)** is denoted with red color. Number of cells; n = 55. DHT: dihydrotestosterone.

Thus, with the use of a LV200 bioluminescent microscope, the impact of a treatment can be visualized and measured by determining the initial and final luminescence status of a single cell (Figure 5A-B). Indeed, change in luminescence over time can be determined by calculating the slope between luminescence measurements from the same cell (Figure 5C). Thus, single cells can be represented in a two-dimensional array with change in luminescence over time and the final luminescence activity as two distinct parameters (Figure 5D). Androgen activation, as measured by luminescence activity, follows a log-normal curvature 37. Data were therefore log-transformed. This representation shows the single-cell AR activity with heterogeneous response to DHT in LAPC4 cells expressing a wild-type AR (Figure 5D). Interestingly, despite being treated with DHT, some cells had a decrease in luminescence activity over time; this demonstrated the complexity of heterogeneous analysis to discriminate non-responsive over responsive cell populations. For that purpose, we decided to use a linear discriminate approach described below to distinguish responsive from non-responsive cell populations.

### PCA3-Cre-PSEBC-ITSTA and dynamic bioluminescence microscopy can assess cell line response to antiandrogen therapy

**Figure 6:**
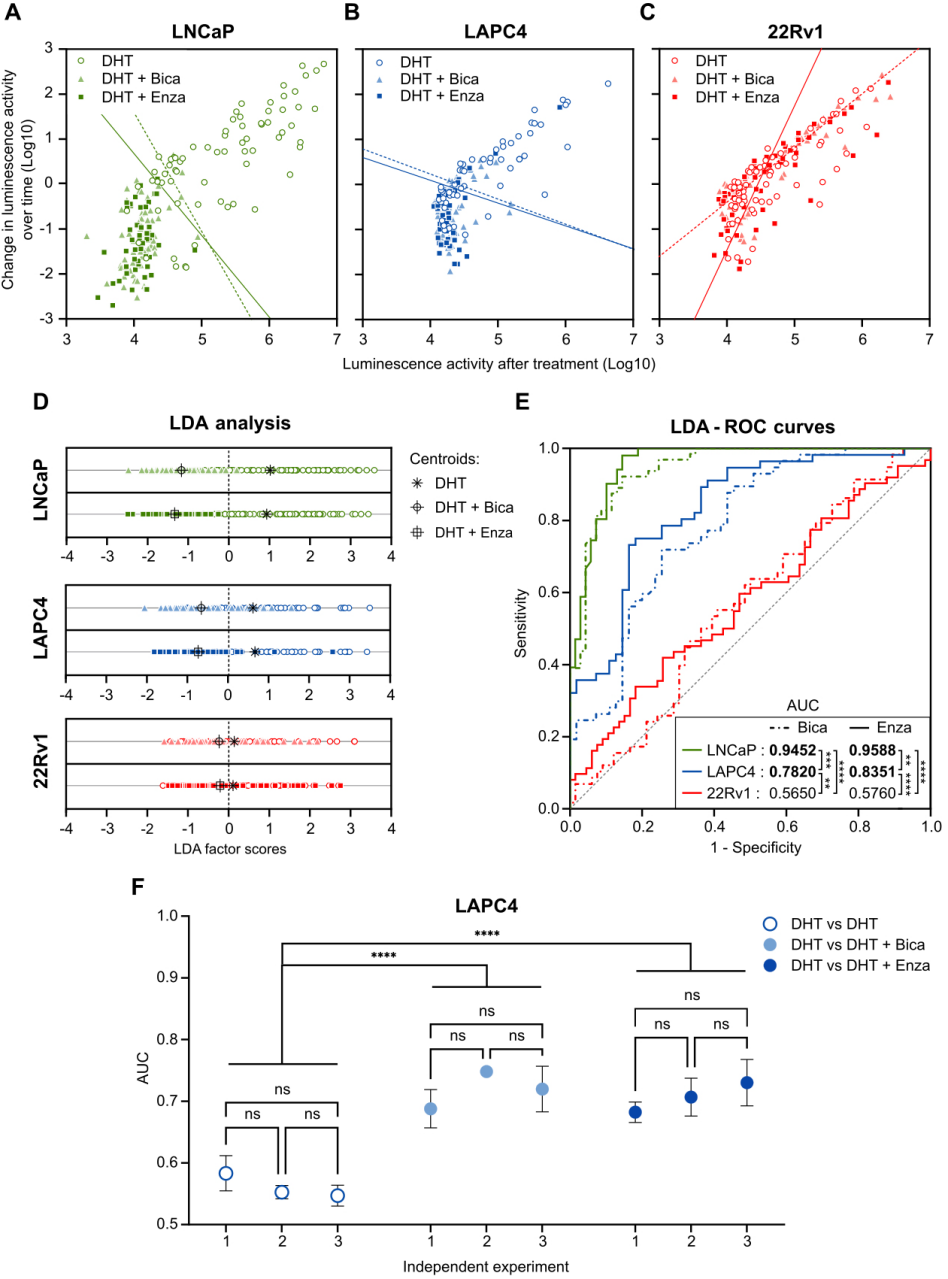
***PCA3*-Cre-*PSEBC*-ITSTA assess single-cell population response to antiandrogen treatment of prostate cancer cells.** Plot of change in luminescence activity *versus* luminescence activity per cell after 48 h of treatment with DHT, DHT + Bica or DHT + Enza in LNCaP (**A**), LAPC4 (**B**) or 22Rv1 (**C**) cells expressing *PCA3*-Cre-*PSEBC*-ITSTA. Dotted and full lines represent LDA decision boundaries of DHT vs DHT + Bica or DHT vs DHT + Enza groups, respectively. (**D**) Charts representing the observations of each cell factor scores after dimensionality reduction by LDA. Upper and bottom chart sections of each cell line compare DHT with DHT + Bica and DHT with DHT + Enza groups, respectively. Black symbols represent the score mean (centroid) for each group. (**E**) ROC curves obtained after performing LDA for each combination of treatment vs DHT data sets. Treated groups with DHT + Bica or DHT + Enza are shown with dot or full lines, respectively. The calculated AUC values for each treatment are indicated in the table inset. AUC values in bold are different from 0.5 (a random classifier) with a *P* value ≤ 0.05. Table inset shows p values of AUC pairwise comparisons using DeLong's method (*p < 0.05, **p < 0.01, ****p < 0.0001). Number of cells analyzed for DHT, DHT + Bica or DHT + Enza treatment with LNCaP cells (n = 69, 64, 51), LAPC4, (n = 55, 45, 56) and 22Rv1 (n = 66, 57, 62), respectively. **(F)** The AUC obtained from linear discriminant analysis are replicable and can discriminate antiandrogen treated from untreated sensitive single-cell populations. Around 75 cells per well for each treatment arm (three wells per treatment arm per experiment) were included in the LDA analysis to build ROC curves and obtain AUC data. Three independent experiments are reported on the x axis. AUC data between DHT and the other treatment arms were compared by analysis of variance (ANOVAs) with post hoc Dunnett's multiple comparison test to determine if there is statistical significance or not. ANOVA with Tukey's multiple comparison test was used to determine if independent experiments were statistically different from each other within the same treatment arm. AUC: area under the curve; Bica: bicalutamide; DHT: dihydrotestosterone; Enza; enzalutamide; LDA: linear discriminant analysis.

The ability of the *PCA3*-Cre-*PSEBC*-ITSTA system to assess the sensitivity of LNCaP (sensitive), LAPC4 (moderately resistant) and 22Rv1 (resistant) PCa cell lines to two ARATs, Enza and Bica 38, 39, was evaluated using a linear discriminate approach. First, change in bioluminescent activity, calculated from each individual cell, was plotted according to bioluminescent activity for each cell after treatment per treatment condition. As seen in Figure 6, treatment of LNCaP or LAPC4 cells with DHT, in combination with Enza or Bica, for 48 h significantly decreased change in bioluminescent activity both over time and after treatment compared to the DHT alone (control condition; Figure 6A-B). In contrast, when the same conditions were applied to ARAT-resistant 22Rv1 cells, no differences were observed in luminescence activity over time or after treatment, demonstrating that resistant cells maintain normal AR signaling when subjected to inefficacious ARAT (Figure 6C). Furthermore, we performed a linear discriminant analysis (LDA) for each combination of treatment (Enza or Bica) and control (DHT) data sets. LDA projects the two-dimensional change in bioluminescent activity over time versus bioluminescent activity after treatment into a single axis that best separates two populations (Figure 6D) and gives an LDA score for each cell. To quantify the difference of the two single-cell populations, we modeled the ROC curves based on the LDA score of each cell per combination of treatment (Enza or Bica) and control (DHT) data sets to calculate the area under the curve (AUC), which here is a synthetic index of the ARAT sensitivity of a single-cell population 13. For example, an AUC of 0.5 reflects an ARAT resistant population because no discrimination could be observed in the AR activity between ARAT-treated and untreated cells.

As expected with the ARAT sensitive LNCaP cell line, the AUC of the ROC curves are close to 1 for both Bica (AUC= 0.9452) and Enza (AUC= 0.9588) -treated cells, meaning the AR activity of single cells differed markedly between LNCaP cells treated with ARAT and control. In contrast, AUCs for the Bica and Enza conditions tested on 22Rv1 cells are 0.5650 and 0.5760, respectively, indicating no distinguishable AR activity between ARAT-treated and untreated 22Rv1 cells, consistent with the ARAT resistant status of 22Rv1 cell line. The AUC for LAPC4 was intermediate for Bica (AUC= 0. 7820) and Enza (AUC= 0. 8351) -treated cells. For all cell lines tested, no significant differences between Enza and Bica ROC curves were observed. However, the AUC of each cell line was different from each other, regardless of the condition tested in compliance with the ARAT status of each cell line (Figure 6E).

To ensure that the data generated was reliable, we tested three independent experiments for LAPC4 cells and observed negligible variation in AUC results (Figure 6F). Moreover, as an internal control, LDA and subsequent ROC curves were generated for combinations of two DHT control data sets. As expected, AUC for these combination conditions were not significantly different from 0.5, consistent with DHT populations behaving like other DHT controls (Figure 6F). This data demonstrates that the *PCA3*-Cre-*PSEBC*-ITSTA system could identify the ARAT sensitivity of single PCa cell populations.

### PCA3-Cre-PSEBC-ITSTA allows dynamic imaging of primary PCa cells from naive PCa patient samples and can evaluate their ARAT sensitivity

**Figure 7:**
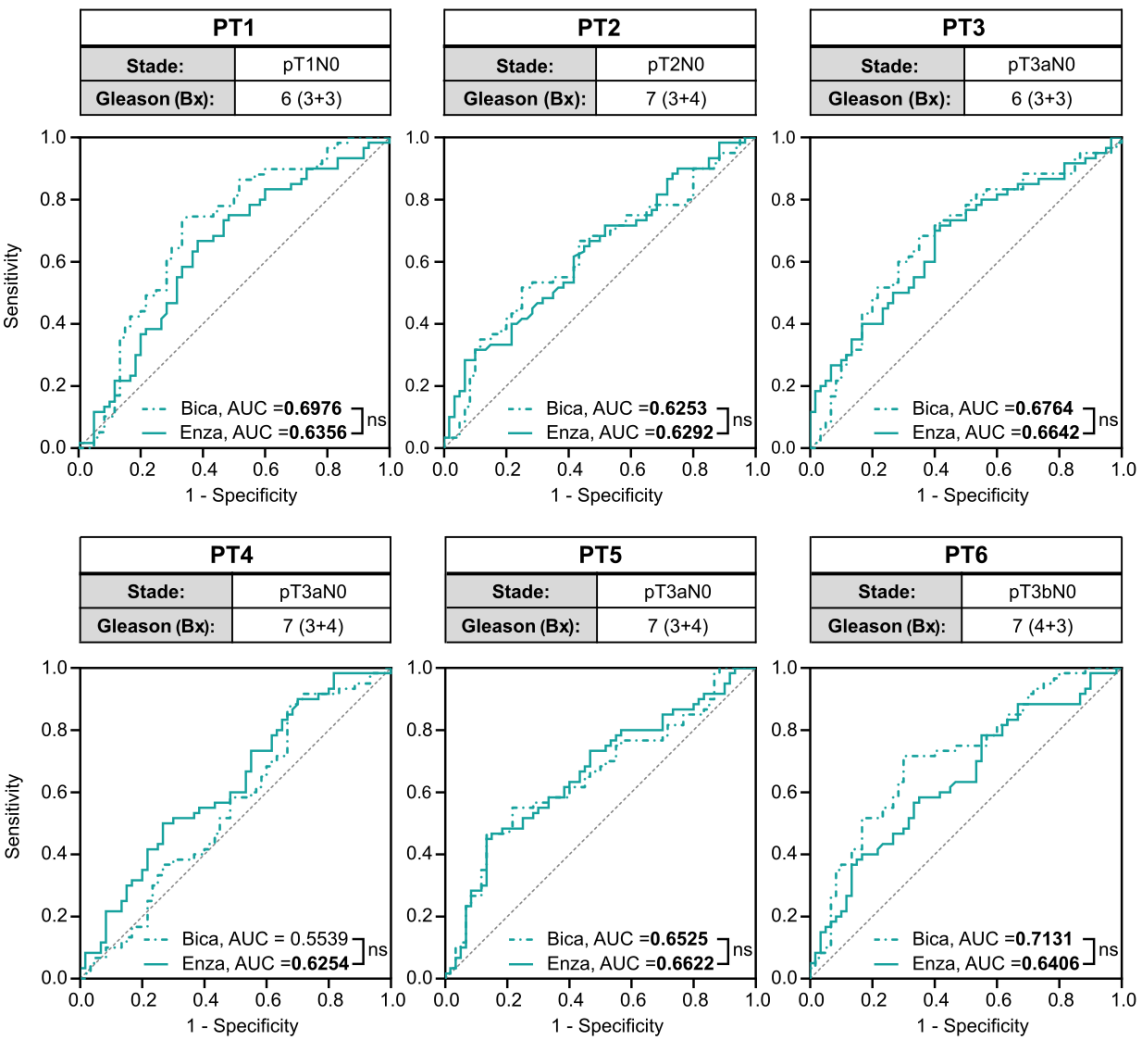
** The *PCA3*-Cre-*PSEBC*-ITSTA system highlights the antiandrogen therapeutic sensitivity in naive primary prostate cancer patient samples.** Clinical information of each patient and ROC curves obtained after performing LDA for each combination of treatment vs DHT control data sets for each naive primary tumor sample. Analyzed cells treated with DHT + Bica or DHT + Enza, are shown with dotted or full line, respectively. AUC values in bold are different from 0.5 (a random classifier) with a *P* value ≤ 0.05. The non-significative difference between AUC is based on Delong method. Number of measured cells for each condition, n = 60. AUC: area under the curve; Bica: bicalutamide; DHT: dihydrotestosterone; Enza; enzalutamide; LDA: linear discriminant analysis.

To investigate the translational potential of the methods, we tested the *PCA3*-Cre-*PSEBC*-ITSTA system on primary cells harvested from prostate specimens of six treatment-naive PCa patients with a Gleason score of 7 or less. Before ARAT, AR active PCa cells were detected in every freshly dissociated sample. Single-cell analysis for each ARAT condition was compared to control DHT to obtain AUC data after LDA, as described above. Average AUC for the Bica and Enza conditions tested on primary cells were significantly different from 0.5, with a mean of 0.6561 and 0.6429, respectively, which correlate with the ARAT naive status of PCa patients. For all samples tested, no significant difference between the Enza and Bica ROC curves were observed (Figure 7). These two observations confirm the ability of the *PCA3*-Cre-*PSEBC*-ITSTA system to identify PCa cells and assess their ARAT sensitivity.

### ARAT therapeutic sensitivity of single PCa cell population from mCRPC patients determined by PCA3-Cre-PSEBC-ITSTA system correlates with clinical patient response

As another step towards clinical translation, we tested the ability of the *PCA3*-Cre-*PSEBC*-ITSTA to identify ARAT-resistance status from primary PCa cells, shedding from the prostate into urine of mCRPC patients with established therapeutic ARAT sensitivity. First, to ensure that luminescent cells detected by *PCA3*-Cre-*PSEBC*-ITSTA system were PCa cells, as opposed to inflammatory, urothelial or benign prostate cells, we stained positive cells with a panel of markers known to distinguish PCa from non-PCa cells 24. Triple PCa marker immunofluorescence (nucleolin, AMACR and NKX3.1) and DAPI staining after cell imaging and fixation showed co-localization of the three signals with that of Luc expressing cells. Several established PCa and bladder cancer cell lines showing that our panel of markers was specific for PCa cells were used as controls (Figure S2).

**Figure 8:**
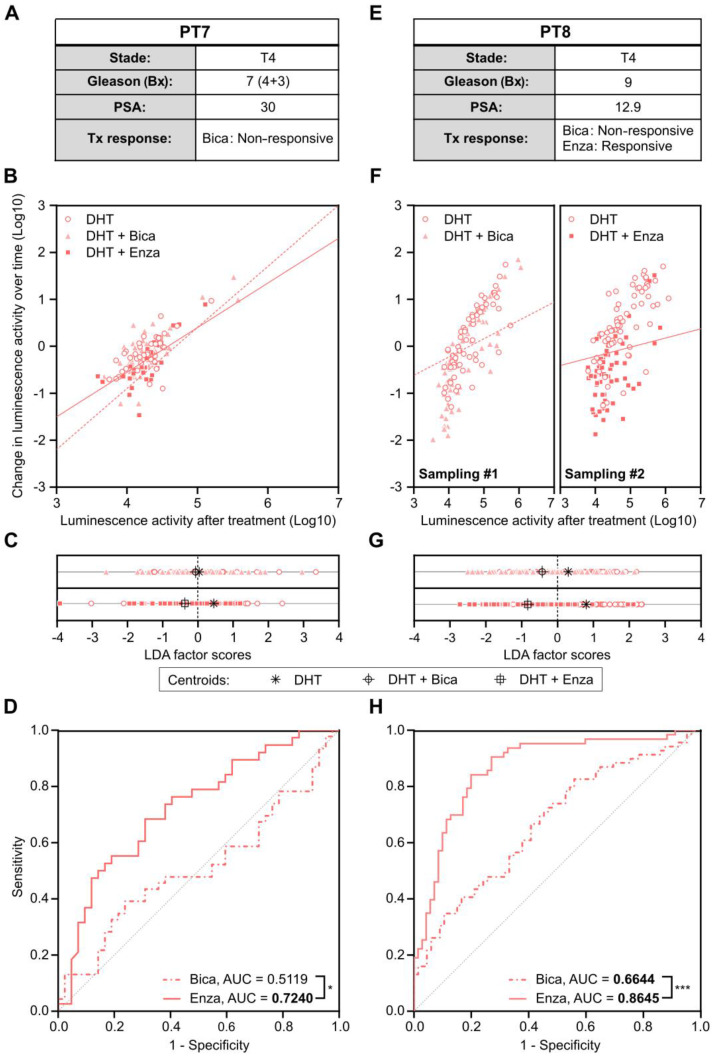
** Antiandrogen sensitivity of single prostate cancer cells population from metastatic castration resistant prostate cancer patients determined by *PCA3*-Cre-*PSEBC*-ITSTA system correlates with clinical patient responses. (A, E)** Clinical information of each patient. **(B, F)** Plots of change in luminescence activity *versus* luminescence activity after treatment for each PCa cells from patient samples after 48 h of treatment with DHT, DHT + Bica or DHT + Enza. Dotted and full lines represent LDA decision boundaries of DHT vs DHT + Bica or DHT vs DHT + Enza groups, respectively. **(C, G)** Charts representing the observations of each cell factor scores after dimensionality reduction by LDA. Upper and bottom chart sections of each cell line compare DHT with DHT + Bica and with DHT + Enza group, respectively. Black symbols represent the score mean (centroid) for each group. (D, H) ROC curves obtained after performing LDA for each combination of treatment vs DHT data sets for each patient sample. Analyzed cells treated with DHT + Bica or DHT + Enza, are shown with dotted or full line, respectively. AUC is calculated for each treatment. AUC values in bold are significantly different (*P* value ≤ 0.05) from 0.5 (a random classifier). Significant differences between AUC are calculated using Delong method (*p < 0.05, ***p < 0.001). The number of measured cells for DHT, DHT + Bica or DHT + Enza condition are respectively; PT7, n = 42, 46, 38 and PT8 sampling #1, n = 65, 69; sampling #2, n = 69, 63. AUC: area under the curve; Bica: bicalutamide; DHT: dihydrotestosterone; Enza; enzalutamide; LDA: linear discriminant analysis.

Metastatic CRPC Patient 7 had progressed after several lines of therapy including Bica, docetaxel and abiraterone acetate before urine sampling. He had previously been treated with enzalutamide for a short duration (less than one month), which was discontinued due to intolerance. Therefore, the patient was not considered enzalutamide-resistant (Figure 8A). Cells collected from urine were isolated and infected with* PCA3*-Cre-*PSEBC*-ITSTA for 72 h. At baseline, 141 positive cells were detected and then treated with either DHT, DHT + Bica, or DHT + Enza for 48 h before reimaging. Change in luminescence activity over time and luminescence activity after treatment was evaluated to generate ROC curves after LDA analysis, as described above (Figure 8B-D). As expected for Bica, an AUC of 0.5119 was obtained, indicating a non-responsive PCa cell population for this treatment. Furthermore, the AUC of 0.7240 for Enza suggested a better clinical efficiency of Enza as an ARAT treatment for this patient (Figure 8D).

Metastatic CRPC Patient 8 initially presented with a high-risk PCa (stage 4 Gleason score 9) and started bicalutamide upon PSA progression under castration. Despite this treatment, the patient's PSA level increased over time, suggesting that the cancer was resistant to bicalutamide. After a switch to enzalutamide, there was a strong PSA drop which showed sensitivity to enzalutamide (Figure 8E). Cells collected from the patient's urine were sampled twice and infected with *PCA3*-Cre-*PSEBC*-ITSTA in the presence of DHT. Baseline bioluminescence imaging was performed 72 h after infection, detecting 281 and 275 cells for the first and second sampling, respectively. After the baseline imaging, cells were then treated with either DHT alone or in combination with Bica or Enza for the first and second sampling, respectively. Single-cell bioluminescence was measured again 48 h post-treatment. The change in luminescence activity over time and luminescence activity after treatment was measured. As seen in Figure 8F-left, we observed an overlap of Bica and DHT treated cell populations when analyzed with these two parameters. In comparison, Enza treated cells showed an apparent reduction in luminescence activity parameters compared to DHT control (Figure 8F-right). Moreover, after LDA analysis, centroids were further apart between Enza and DHT conditions than between Bica and DHT conditions (Figure 8G). The corresponding ROC curves generated after LDA analysis demonstrated that the ability to discriminate single cells between untreated and treated groups was significantly better with Enza than Bica (Figure 8H). The AUC obtained for Bica and Enza were 0.6644 and 0.8645 respectively and in concordance with the observed Bica clinical response (Figure 8E and H). Overall, these results demonstrated that single-cell bioluminescence microscopy extracted data could serve as predictive response biomarkers to ARAT.

## Discussion

In this study, we show that single cancer cell drug response can be monitored and integrated by transcription-based luminescence biosensors using bioluminescence microscopy to determine the drug sensitivity of a cell population. As proof of principle, we have developed and validated our method in primary and cell line-derived prostate cancers cells. Prostate cancer cells were transduced by our prostate cancer-specific biosensor and cultured; the target (androgen receptor activity) was monitored in real-time upon exposure to an anticancer drug (antiandrogen). Using this novel quantitative method, we were able to detect primary prostate cancer cells and determine their overall dynamic individual response to ARAT. Using linear discriminate analysis (LDA), we were able to determine ARAT sensitivity levels in several cell-lines or patient-derived cell populations. We show that the technology described above could represent a new way to monitor patient's cell population drug sensitivity and act as a novel predictive biomarker.

With disease progression under treatment pressure, it has been shown that genotypic characteristics of cells are heterogeneous and plastic 40, 41. Improvements in the clinical outcomes of many cancer types are likely to be achieved by giving patients a drug tailored to the genetic makeup of their tumor. Biomarkers predicting therapeutic responses are frequently evaluated on tumor biopsy samples, which incorporate bulk analysis of whole tissue samples 42. This results in response-predictive biomarker panels built based on the presence or absence of resistance or response genotypes. Therefore, this approach dichotomizes the prediction of response as a “candidate” or “not a candidate” to a drug based on the “detected” or “not detected” genomic alteration. Contrary to static biomarkers, our method has the potential to determine sensitivity as a continuous variable rather than a discrete variable. The clinical translation of a biomarker that establishes a continuous probability of response is the ability to guide clinicians towards therapy intensification, rather than to opt for another therapy that would also miss a cancer cell subpopulation. For instance, in the PCa cell lines tested using our methods (Figure 6), we could postulate that a patient with the LNCaP response profile would be a better candidate for androgen deprivation therapy (ADT) treatment alone; meanwhile, a patient with an LAPC4 profile might benefit from combination therapy of ADT and docetaxel (chemotherapy). Similarly, a patient with a 22Rv1 antiandrogen response profile would be a better candidate for a non-ADT-based treatment, such as chemotherapy alone. Indeed, bulk tissue biomarkers may mislead clinicians to drug selection that would have a minor impact on disease. This mitigated response can be explained by the bulk tissue detection of the predictive biomarker in a minor subpopulation of sensitive cancer cells, while another resistant subclone could progress until clinical progression is detected and treatment changed. Similarly, some treatments may be disregarded as a result of the detection of resistance genotypes in a minority of cells, while the majority of the cancer cells would have been sensitive to treatment and the patient could have responded. Therefore, single-cell population quantitative analysis may become a key method used to direct patients towards the best treatment combinations or sequences.

The method presented here, which is based on drug-target single-cell imaging, has a unique ability to be highly integrative at the molecular, cellular and cell population (tumor) levels. It can detect AR pathway molecular alterations by monitoring the activity of AR in real time. Through dynamic imaging of single-cell AR activity upon ARAT exposure, this method integrates most ARAT resistance mechanisms (resistome) and their interactions together to escape from ARAT inhibitory effects. Our single-cell analysis using *PCA3*-Cre-*PSEBC*-ITSTA may provide a better characterization of the response to ARAT in PCa patients compared to bulk analysis, given the recent literature on intercellular and even intracellular PCa cell genotypic alteration and heterogeneity 21, 43. As opposed to organoids or patient-derived xenografts, the shorter-term duration of cell culture needed in our method can help to avoid differentiation and selection associated with the *ex-vivo* environment 44-46. Moreover, in our methods, cells remain viable and each single cell can be analyzed independently and harvested for molecular analysis after treatment exposure. Therefore, live single-cell imaging phenotypes, sensitive or resistant, can be linked to omics analysis to better understand the genomic, transcriptomic and proteomic parameters involved in drug resistance. We believe that such a reconstructive approach from single molecular alterations to whole tumor biology has great potential because it has been previously shown that freshly dissociated cells from the tumor mirror the genotypic characteristics of the tumor 24, 25. This method is also versatile because various sources of samples such as biopsies, blood, peritoneal (ascites), urine, pleural or cerebrospinal fluids can be exploited to harvest cancer cells 25, 47-50. Moreover, in prostate cancer, the anatomical position of the prostate makes prostate biopsies and urine important sources of cancer cells, which could facilitate the translation of the technology into the clinics.

As a proof of concept, we confirmed the ability of our system to correctly define response to ARATs like enzalutamide at the single-cell level, using human PCa cell lines with known drug sensitivity. When tested on different cohorts of patients from primary PCa to mCRPC, our method could discriminate the overall response of a patient to antiandrogens. In cases of defined sensitivity, we detected a significant decrease in AR activity following exposure to conventional ARAT in naive primary PCa cells. Moreover, in both cases of mCRPC patients, androgen response established with *PCA3*-*Cre*-*PSEBC*-ITSTA correlated with the clinical ARAT sensitivity status.

Our method has some limitations. First, the cell dissociation procedure and cell culture conditions could affect the viability of primary prostate cancer cells. However, with the LDA-based analysis method, the single-cell population that is tested is always compared to a DHT control population under the same conditions, which compensate for non-specific cell death, viral transduction and other technical factors, independent of the treatment. Secondly, like any cell targeting imaging method relying on specific genes expression, this method relies on specific promoter activation which might not be expressed in all cancer cells, implying that some cancer cells would not be detected. For instance, AR-negative PCa cells are not imaged with the system presented herein, but this population is not targeted by ARAT. Finally, intrapatient intermetastasis polyclonality has been described in advanced PCa, which might limit the predictive value of single-site biopsies 43, 51. However, this is also a limitation for any biopsy-based biomarker approach and it seems that this clinical situation is found in a limited number of patients 52, 53.

## Conclusion

We show that single cancer cell drug response can be monitored and integrated by transcription-based luminescence biosensors using bioluminescence microscopy to determine the drug sensitivity of a cell population. The *PCA3*-Cre-*PSEBC*-ITSTA system, which is based on combinational activation of two prostate cancer gene promoters, has the ability to study dynamic and quantitative antiandrogen single-cell response from urine and primary cancer tissues harvested from PCa patients. This novel method could be expanded to other cancer types by using tissue specific promoters along with regulatory elements for drug targets and act as a predictive biomarker method for several cancer treatments.

## Supplementary Material

Supplementary figures and table.

## Abbreviations

3STA: three-step transcriptional amplification system
ADT: androgen deprivation therapy
AMACR: alpha-Methylacyl-CoA racemase
AR: androgen receptor
ARAT: androgen receptor-axis-targeted therapies
AUC: area under the curve
*BGH-Ig*: human β-globin chimeric intron
BGH: bovine growth hormone
Bica: bicalutamide
BSA: bovine serum albumin
CRPC: castration-resistant prostate cancer
DAPI: 4-6-diamidino-2-phenylindole
DHT: dihydrotestosterone
Enza: enzalutamide
FBS: fetal bovine serum
FBS-CT: charcoal treated fetal bovine serum
FOV: field of view
GFP: green fluorescent protein
ivp: infectious viral particles
LDA: linear discriminate analysis
Luc: firefly luciferase
mCRPC: metastatic castration-resistant prostate cancer
MOI: multiplicity of infection
MP-ITSTA: multi-promoter integrated two-step transcriptional amplification system
PCa: prostate cancer
*Prm2*: mice protamine intron
PSA: prostate specific antigen
RCN: relative copy number
RLU: relative light unit
RT: room temperature
SV40): Simian Virus 40
TSTA: two-step transcriptional amplification system

## References

[1] Watson PA, Arora VK, Sawyers CL (2015). Emerging mechanisms of resistance to androgen receptor inhibitors in prostate cancer. Nat Rev Cancer.

[2] Chandrasekar T, Yang JC, Gao AC, Evans CP (2015). Mechanisms of resistance in castration-resistant prostate cancer (CRPC). Transl Androl Urol.

[3] Dagogo-Jack I, Shaw AT (2018). Tumour heterogeneity and resistance to cancer therapies. Nat Rev Clin Oncol.

[4] Marusyk A, Janiszewska M, Polyak K (2020). Intratumor Heterogeneity: The Rosetta Stone of Therapy Resistance. Cancer Cell.

[5] Cyll K, Ersvaer E, Vlatkovic L, Pradhan M, Kildal W, Avranden Kjaer M (2017). Tumour heterogeneity poses a significant challenge to cancer biomarker research. Br J Cancer.

[6] Mateo J, McKay R, Abida W, Aggarwal R, Alumkal J, Alva A (2020). Accelerating precision medicine in metastatic prostate cancer. Nat Cancer.

[7] Suphavilai C, Chia S, Sharma A, Tu L, Da Silva RP, Mongia A (2020). Predicting heterogeneity in clone-specific therapeutic vulnerabilities using single-cell transcriptomic signatures. bioRxiv. 2020.

[8] McFarland JM, Paolella BR, Warren A, Geiger-Schuller K, Shibue T, Rothberg M (2020). Multiplexed single-cell transcriptional response profiling to define cancer vulnerabilities and therapeutic mechanism of action. Nat Commun.

[9] Dai Z, Gu XY, Xiang SY, Gong DD, Man CF, Fan Y (2020). Research and application of single-cell sequencing in tumor heterogeneity and drug resistance of circulating tumor cells. Biomark Res.

[10] Yuan GC, Cai L, Elowitz M, Enver T, Fan G, Guo G (2017). Challenges and emerging directions in single-cell analysis. Genome Biol.

[11] Spiller DG, Wood CD, Rand DA, White MR (2010). Measurement of single-cell dynamics. Nature.

[12] Jeknic S, Kudo T, Covert MW (2019). Techniques for Studying Decoding of Single Cell Dynamics. Front Immunol.

[13] Cetin AE, Stevens MM, Calistri NL, Fulciniti M, Olcum S, Kimmerling RJ (2017). Determining therapeutic susceptibility in multiple myeloma by single-cell mass accumulation. Nat Commun.

[14] Calistri NL, Kimmerling RJ, Malinowski SW, Touat M, Stevens MM, Olcum S (2018). Microfluidic active loading of single cells enables analysis of complex clinical specimens. Nat Commun.

[15] Jain P, Neveu B, Velot L, Wu L, Fradet Y, Pouliot F (2016). Bioluminescence Microscopy as a Method to Measure Single Cell Androgen Receptor Activity Heterogeneous Responses to Antiandrogens. Sci Rep.

[16] Azad T, Janse van Rensburg HJ, Lightbody ED, Neveu B, Champagne A, Ghaffari A (2018). A LATS biosensor screen identifies VEGFR as a regulator of the Hippo pathway in angiogenesis. Nat Commun.

[17] Sasportas LS, Hori SS, Pratx G, Gambhir SS (2014). Detection and quantitation of circulating tumor cell dynamics by bioluminescence imaging in an orthotopic mammary carcinoma model. PLoS One.

[18] Jain P, Clermont P-L, Desmeules F, Zoubeidi A, Neveu B, Pouliot F (2019). Development of a Transcriptional Amplification System Based on the PEG3 Promoter to Target Androgen Receptor-Positive and -Negative Prostate Cancer Cells. International journal of molecular sciences.

[19] Yeh HW, Ai HW (2019). Development and Applications of Bioluminescent and Chemiluminescent Reporters and Biosensors. Annu Rev Anal Chem (Palo Alto Calif).

[20] Mazo-Vargas A, Park H, Aydin M, Buchler NE (2014). Measuring fast gene dynamics in single cells with time-lapse luminescence microscopy. Mol Biol Cell.

[21] Miyamoto DT, Zheng Y, Wittner BS, Lee RJ, Zhu H, Broderick KT (2015). RNA-Seq of single prostate CTCs implicates noncanonical Wnt signaling in antiandrogen resistance. Science.

[22] Schalken JA, Hessels D, Verhaegh G (2003). New targets for therapy in prostate cancer: differential display code 3 (DD3(PCA3)), a highly prostate cancer-specific gene. Urology.

[23] Neveu B, Jain P, Tetu B, Wu L, Fradet Y, Pouliot F (2016). A PCA3 gene-based transcriptional amplification system targeting primary prostate cancer. Oncotarget.

[24] Fujita K, Pavlovich CP, Netto GJ, Konishi Y, Isaacs WB, Ali S (2009). Specific detection of prostate cancer cells in urine by multiplex immunofluorescence cytology. Hum Pathol.

[25] Nickens KP, Ali A, Scoggin T, Tan SH, Ravindranath L, McLeod DG (2015). Prostate cancer marker panel with single cell sensitivity in urine. Prostate.

[26] Goodwin EC, Rottman FM (1992). The 3'-flanking sequence of the bovine growth hormone gene contains novel elements required for efficient and accurate polyadenylation. J Biol Chem.

[27] Kaczmarczyk SJ, Green JE (2001). A single vector containing modified cre recombinase and LOX recombination sequences for inducible tissue-specific amplification of gene expression. Nucleic Acids Res.

[28] Gu H, Zou YR, Rajewsky K (1993). Independent control of immunoglobulin switch recombination at individual switch regions evidenced through Cre-loxP-mediated gene targeting. Cell.

[29] Evans RK, Nawrocki DK, Isopi LA, Williams DM, Casimiro DR, Chin S (2004). Development of stable liquid formulations for adenovirus-based vaccines. J Pharm Sci.

[30] Thorne N, Inglese J, Auld DS (2010). Illuminating insights into firefly luciferase and other bioluminescent reporters used in chemical biology. Chem Biol.

[31] DeLong ER, DeLong DM, Clarke-Pearson DL (1988). Comparing the areas under two or more correlated receiver operating characteristic curves: a nonparametric approach. Biometrics.

[32] Hoess R, Wierzbicki A, Abremski K (1985). Formation of small circular DNA molecules via an *in vitro* site-specific recombination system. Gene.

[33] Kwon YK, Hecht NB (1993). Binding of a phosphoprotein to the 3' untranslated region of the mouse protamine 2 mRNA temporally represses its translation. Mol Cell Biol.

[34] Palmer AC, Egan JB, Shearwin KE (2011). Transcriptional interference by RNA polymerase pausing and dislodgement of transcription factors. Transcription.

[35] Ray S, Paulmurugan R, Patel MR, Ahn BC, Wu L, Carey M (2008). Noninvasive imaging of therapeutic gene expression using a bidirectional transcriptional amplification strategy. Mol Ther.

[36] Sato M, Figueiredo ML, Burton JB, Johnson M, Chen M, Powell R (2008). Configurations of a two-tiered amplified gene expression system in adenoviral vectors designed to improve the specificity of *in vivo* prostate cancer imaging. Gene Ther.

[37] Pouliot F, Rouleau M, Neveu B, Toren P, Morin F, Velot L (2020). Extragonadal Steroids Contribute Significantly to Androgen Receptor Activity and Development of Castration Resistance in Recurrent Prostate Cancer after Primary Therapy. J Urol.

[38] Kregel S, Wang C, Han X, Xiao L, Fernandez-Salas E, Bawa P (2020). Androgen receptor degraders overcome common resistance mechanisms developed during prostate cancer treatment. Neoplasia.

[39] Cunningham D, You Z (2015). *In vitro* and *in vivo* model systems used in prostate cancer research. J Biol Methods.

[40] Buettner F, Natarajan KN, Casale FP, Proserpio V, Scialdone A, Theis FJ (2015). Computational analysis of cell-to-cell heterogeneity in single-cell RNA-sequencing data reveals hidden subpopulations of cells. Nat Biotechnol.

[41] Qin S, Jiang J, Lu Y, Nice EC, Huang C, Zhang J (2020). Emerging role of tumor cell plasticity in modifying therapeutic response. Signal Transduct Target Ther.

[42] Gaudreau PO, Stagg J, Soulieres D, Saad F (2016). The Present and Future of Biomarkers in Prostate Cancer: Proteomics, Genomics, and Immunology Advancements. Biomark Cancer.

[43] Haffner MC, Zwart W, Roudier MP, True LD, Nelson WG, Epstein JI (2021). Genomic and phenotypic heterogeneity in prostate cancer. Nat Rev Urol.

[44] Shi J, Li Y, Jia R, Fan X (2020). The fidelity of cancer cells in PDX models: Characteristics, mechanism and clinical significance. Int J Cancer.

[45] Liu C, Qin T, Huang Y, Li Y, Chen G, Sun C (2020). Drug screening model meets cancer organoid technology. Transl Oncol.

[46] Lynch M, Ackerman MS, Gout JF, Long H, Sung W, Thomas WK (2016). Genetic drift, selection and the evolution of the mutation rate. Nat Rev Genet.

[47] Ahmed N, Greening D, Samardzija C, Escalona RM, Chen M, Findlay JK (2016). Unique proteome signature of post-chemotherapy ovarian cancer ascites-derived tumor cells. Sci Rep.

[48] Puglisi M, Stewart A, Thavasu P, Frow M, Carreira S, Minchom A (2016). Characterisation of the Phosphatidylinositol 3-Kinase Pathway in Non-Small Cell Lung Cancer Cells Isolated from Pleural Effusions. Oncology.

[49] Wang Y, Springer S, Zhang M, McMahon KW, Kinde I, Dobbyn L (2015). Detection of tumor-derived DNA in cerebrospinal fluid of patients with primary tumors of the brain and spinal cord. Proc Natl Acad Sci U S A.

[50] Andree KC, Mentink A, Zeune LL, Terstappen L, Stoecklein NH, Neves RP (2018). Toward a real liquid biopsy in metastatic breast and prostate cancer: Diagnostic LeukApheresis increases CTC yields in a European prospective multicenter study (CTCTrap). Int J Cancer.

[51] Morin F, Beauregard JM, Bergeron M, Nguile Makao M, Lacombe L, Fradet V (2017). Metabolic Imaging of Prostate Cancer Reveals Intrapatient Intermetastasis Response Heterogeneity to Systemic Therapy. Eur Urol Focus.

[52] Fontugne J, Davis K, Palanisamy N, Udager A, Mehra R, McDaniel AS (2016). Clonal evaluation of prostate cancer foci in biopsies with discontinuous tumor involvement by dual ERG/SPINK1 immunohistochemistry. Mod Pathol.

[53] Kristiansen A, Bergstrom R, Delahunt B, Samaratunga H, Guethjonsdottir J, Gronberg H (2019). Somatic alterations detected in diagnostic prostate biopsies provide an inadequate representation of multifocal prostate cancer. Prostate.

