# Supplementary material for: A transcriptional biosensor to monitor single cancer cell therapeutic responses by bioluminescence microscopy
Source: Theranostics. 2022 Jan 1;12(2):474–92. doi: 10.7150/thno.63744 (PMC8692902; doi:10.7150/thno.63744)
Supplement: Supplementary file 1 — Supplementary figures and table. [file thnov12p0474s1.pdf]

## Supplemental Table

### Table S1: Primers for plasmid constructions and RT-qPCR

| Amplicons | Directions | Sequences |
|---|---|---|
| **Cre fragment 1-465 pb** | Forward | GGGGAGATTTGTGTGGGTCGACACCATGCCCAAGAAG |
| | Reverse | GTAACCTTGATACTTACACCTGGTCGAAATCAGTGCGTTC |
| **Cre fragment 466-1523 pb** | Forward | TCGTTCACTCATGGAAAATAGCG |
| | Reverse | GTACAAGAAAGCTGGGTAAAGCTTGTCCGCCACACCCAG |
| **Intron BGH-Ig** | Forward | GTAAGTATCAAGGTTACAAGACAGGTTTAAG |
| | Reverse | CTATTTTCCATGAGTGAACGACTGTGGAGAGAAAGGCAAAGTG |
| **Intron Prm2** | Forward | AAGTAGAGGGCTGGGCTG |
| | Reverse | CCATGAGTGAACGAACCTAGAAAGGTAAGAAAAGTG |
| **Intron Prm2-AG** | Forward | GTAGAGGGCTGGGCTGGGC |
| | Reverse | CCATGAGTGAACGAACCTAGAAAGGTAAGAAAAGTG |
| ***PSEBC*** | Forward | GGATCCGTCGAATTTAAATAAATCTAGCTGATATAGTGTGGC |
| | Reverse | ATACGAAGTTATTGCGCAGGCTGGGGAGCCTCCCCCAG |
| **Primer set 1 (Luc)** | Forward | TATCTCTTCATAGCCTTATGCA |
| | Reverse | GGTAAAGCCACCATGGAAGA |
| | Probe | AGGCCCGGCGCCATTCTATCCGCTGGA |
| **Primer set 2 (stop cassette)** | Forward | GTGCCTTCTAGTTGCCAG |
| | Reverse | ATAGAATGACACCTACTCAGACA |
| | Probe | TGCCCCTCCCCCGTGCCTTCCTTGA |

**Supplemental Figures and Legends**

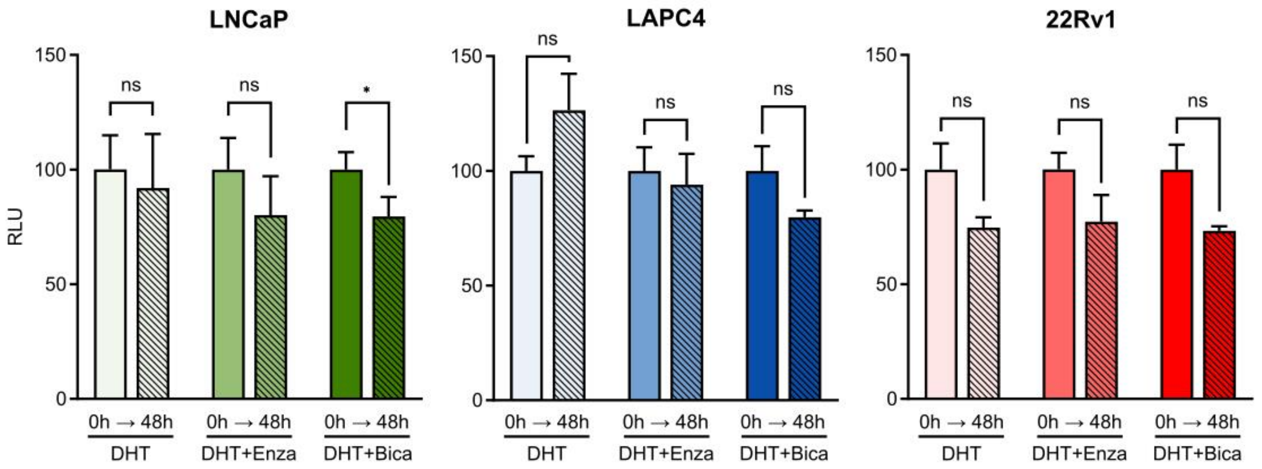


**Figure S1. Bioluminescent plate reader cannot monitor dynamic prostate cancer cell line antiandrogen sensitivity after *PCA3*-Cre-*PSEBC*-ITSTA transduction.** Luciferase activity was measured in the same wells, before and after 48 h of treatment (DHT, DHT + Bica or DHT + Enza) of ARAT responsive (LNCaP and LAPC4) and non-responsive (22Rv1) prostate cancer cells infected with *PCA3*-Cre-*PSEBC*-ITSTA. The luciferase activity is represented as relative activity over initial measurement (t = 0 h). The data represents mean of triplicates ± S.D. Data were compared by paired Student's t-test. ARAT: androgen receptor-axis-targeted therapies; Bica: bicalutamide; DHT: dihydrotestosterone; Enza: Enzalutamide; ns: non significative; RLU: relative light unit.

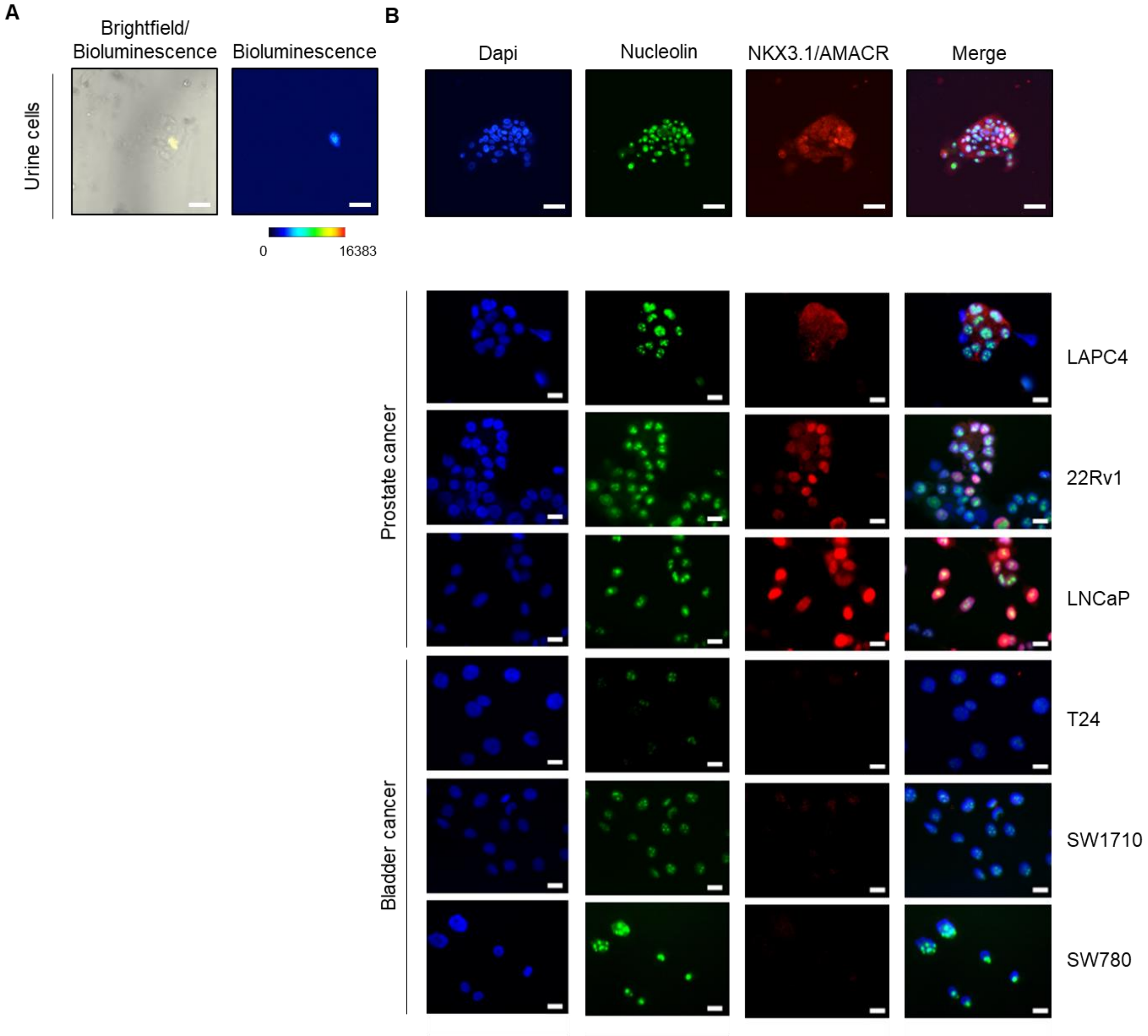


**Figure S2. Immunofluorescence staining with multiple markers confirms the prostate cancer origin of urine cells expressing bioluminescence.** (**A**) Bioluminescence imaging of urine cells expressing *PCA3*-Cre-*PSEBC*-ITSTA system. (**B**) Immunofluorescence staining with multiple markers DAPI, Nucleolin, NKX3.1 and AMACR showed higher expression and were co-localized in prostate cancer cells. After bioluminescence imaging of cells collected from urine or 24 hours after cell line seeding, the cells were fixed in paraformaldehyde. The cells were exposed to antibodies against Nucleolin, NKX3.1 and AMACR as well as stained for DAPI. The wells were then imaged at 40X magnification using fluorescence microscope. Scale bars represent 50 μm for cells collected from urine and 20 μm for cell lines.
